# Anti-Müllerian hormone a surrogate of follicular fluid oxidative stress in polycystic ovary syndrome?

**DOI:** 10.3389/fcell.2024.1408879

**Published:** 2024-07-01

**Authors:** Emídio Vale-Fernandes, Mafalda V. Moreira, Bárbara Rodrigues, Sofia S. Pereira, Carla Leal, Márcia Barreiro, António Tomé, Mariana P. Monteiro

**Affiliations:** ^1^ Centre for Medically Assisted Procreation/Public Gamete Bank, Gynaecology Department, Centro Materno-Infantil do Norte Dr. Albino Aroso (CMIN), Unidade Local de Saúde de Santo António (ULSSA), Porto, Portugal; ^2^ UMIB - Unit for Multidisciplinary Research in Biomedicine, ICBAS - School of Medicine and Biomedical Sciences, University of Porto, Porto, Portugal; ^3^ ITR - Laboratory for Integrative and Translational Research in Population Health, Porto, Portugal; ^4^ Gynaecology Department, Centro Materno-Infantil do Norte Dr. Albino Aroso (CMIN), Unidade Local de Saúde de Santo António (ULSSA), Porto, Portugal; ^5^ i3S - Instituto de Investigação e Inovação em Saúde, Universidade do Porto, Porto, Portugal; ^6^ Molecular Genetics Unit, Centro de Genética Médica Dr. Jacinto Magalhães (CGM), Unidade Local de Saúde de Santo António (ULSSA), Porto, Portugal

**Keywords:** Anti-Müllerian hormone, oxidative stress, follicular fluid, *in vitro* fertilization, polycystic ovary syndrome

## Abstract

Polycystic ovary syndrome (PCOS) is the most common endocrinopathy in women at childbearing age. Anti-Müllerian hormone (AMH) is a widely accepted sensitive marker of ovarian reserve, which has been suggested that could also act as biomarker of ovarian morphology for PCOS diagnosis. Oxidative stress (OS) is known to be associated and have a negative impact factor in several reproductive conditions, including PCOS. However, the relationship between circulating AMH and OS within the follicular fluid (FF), and its potential impact on *in vitro* fertilization (IVF) outcomes of women with PCOS, remains largely unexplored. A total of 84 women, with PCOS (n = 30) or ovulatory controls (n = 54), were enrolled in this study. Women underwent individualized controlled ovarian stimulation for oocyte retrieval. Blood and FF obtained from mature follicles were collected at the time of oocyte retrieval, for measuring total testosterone, ∆4-androstenedione, progesterone, sex hormone binding globulin (SHBG) and AMH. OS in the FF was assessed by measuring total antioxidant capacity (TAC) through the ferric reducing antioxidant power (FRAP) and lipid peroxidation (LPO) by quantification of malondialdehyde (MDA) levels. Our results demonstrated that women with PCOS had significantly higher plasma levels of AMH, ∆4-androstenedione, total testosterone and a free androgen index (FAI) than observed in non-PCOS controls. In women with PCOS, total testosterone and AMH levels in the FF were also higher, while TAC was lower compared to non-PCOS. Furthermore, circulating AMH levels were positively correlated with ∆4-androstenedione, albeit negatively correlated with TAC. In this study we demonstrated that the susceptibility to OS, as assessed by the total antioxidant capacity in the FF, is higher in women with PCOS and inversely related to AMH levels. This study results lead us to forge the reasonable hypothesis that the greater susceptibility to OS within the follicle microenvironment is potentially at the end of a roadway that starts with elevated ∆4-androstenedione and AMH within the FF, which in turn are mirrored by circulating AMH and androgen levels. Thus, suggesting that circulating AMH levels could act as a surrogate biomarker of follicular fluid oxidative stress in women with PCOS.

## 1 Introduction

Polycystic ovary syndrome (PCOS) is the most common endocrinopathy in women at childbearing age with a prevalence ranging from five to 18% depending on the population (Rotterdam ESHRE/ASRM-Sponsored PCOS consensus workshop group, 2004). PCOS diagnosis relies on the Rotterdam criteria, which requires the presence of at least two out of three parameters: irregular menstrual cycles, clinical or biochemical hyperandrogenism and polycystic ovarian morphology (Christ and Cedars, 2023).

Anti-Müllerian hormone (AMH) is a polypeptide secreted by cells of the granulosa layer of primary/preantral ovarian follicles and small secondary/antral follicles (van Rooij et al., 2002). AMH is a widely accepted sensitive marker of ovarian reserve, given that the secretion tends to be proportional to the granulosa cell mass (La Marca et al., 2009; Tal et al., 2014; Vale-Fernandes et al., 2023b). Additionally, AMH has been pointed as a possible surrogate marker of ovarian morphology (Dumont et al., 2015), once it is typically elevated in women with PCOS, as compared to normoovulatory women (Cook et al., 2002). Despite controversial, it has been suggested that AMH should be integrated into the Rotterdam criteria for PCOS diagnosis (Teede et al., 2023). Moreover, there is also evidence that AMH levels may correlate with reproductive outcomes in Assisted Reproductive Technology (ART) procedures, including pregnancy and live birth rates, irrespective of age (Brodin et al., 2013).

PCOS accounts for a considerable proportion of the causes of infertility among couples seeking for ART. Despite most women with PCOS exhibiting positive responses to ovarian stimulation for *in vitro* fertilization (IVF), characterized by a higher-than-average number of growing follicles, their reproductive outcomes often prove less favorable compared to women experiencing infertility from other causes (Rajani et al., 2012; Piomboni et al., 2014; Yilmaz et al., 2016; Artimani et al., 2018). Factors appointed as potentially detrimental to reproductive outcomes in women with PCOS, include the competence of the endometrium (Piltonen, 2016) and the unfavorable follicular microenvironment compromising the physiological functions and quality of oocytes (Qiao and Feng, 2010; Palomba et al., 2017).

Oxidative stress (OS) is recognized as an impacting factor in the pathophysiology of several infertility-related problems, including PCOS. OS occurs when the production of reactive oxygen species (ROS) surpasses the antioxidant capacity. Despite the physiological role of ROS in the modulation of a spectrum of reproductive functions (Shkolnik et al., 2011; Wang et al., 2017; Lu et al., 2018), the disruption of the redox signaling can lead mitochondrial dysfunction, protein oxidation, lipid peroxidation and DNA damage, negatively impacting on reproductive function (Sasaki et al., 2019; Jamil et al., 2020). Previous studies have demonstrated higher levels of pro-oxidant markers and lower levels of antioxidants in the follicular fluid (FF) of women with PCOS when compared to normoovulatory women (Chattopadhayay et al., 2010; Artimani et al., 2018; Naigaonkar et al., 2021; Moreira et al., 2023). Given the complex nature of PCOS and potential deleterious impact of OS on the follicular microenvironment and follicle growth, it is important to deepen the knowledge on the putative triggers of OS in FF. In particular, the relationship between AMH and OS within the FF, and its potential impact on IVF outcomes, remains largely unexplored.

Therefore, to address this gap in knowledge, this study aimed to investigate the association between circulating and FF AMH and OS markers within the FF.

## 2 Materials and methods

### 2.1 Participants’ recruitment

A total of 84 women undergoing *in vitro* fertilization (IVF) were enrolled in this study. Women were allocated into two groups depending on the presence of PCOS (n = 30) or non-PCOS ovulatory controls (n = 54) The diagnosis of PCOS was based on the 2003 Rotterdam diagnostic criteria, which requires the presence of at least two of the following three manifestations: (1) oligo- and/or anovulation, (2) clinical and/or biochemical evidence of hyperandrogenism, and (3) polycystic ovaries on ultrasound examination (at least 10 follicles 2–9 mm in size or volume of the ovary greater than 10 mL). The control group included women who underwent ART treatments for oocyte donation or infertility treatment due to tubal and/or male factors. Exclusion criteria included women with diminished ovarian reserve, endometriosis, systemic diseases, abnormal prolactin levels, and/or thyroid dysfunction. Electronic medical records were used for data extraction, namely, age, body mass index (BMI), hormonal measurements, and IVF cycle characteristics. All patients were recruited to participate and enrolled in the study after providing written informed consent to participate in this study. The study protocol was authorized by the Ethics Committee of the Institution [2020.119 (097-DEFI/099-CE)].

### 2.2 Ovarian stimulation protocol

Women underwent a Gonadotropin-Releasing Hormone (GnRH) antagonist protocol with individualized controlled ovarian stimulation based on ovarian reserve testing and standard clinical practice. Two different types of ovarian stimulation protocols are used for assisted reproduction, the long protocol with a GnRH agonist and the short protocol with GnRH antagonist. However, for safety reasons given the risk of ovarian hyperstimulation syndrome, in women with PCOS only the short protocol with GnRH antagonist, avoiding the trigger with human chorionic gonadotropin (βHCG), is used.

### 2.3 Collection of blood plasma and follicular fluid

Follicular fluid (FF) of each woman was collected at the day of oocyte retrieval. The ovulatory trigger was performed in preparation for aspiration, whenever a minimum of two or three follicles reached the size of 17 mm in diameter. Nevertheless, all follicles were aspirated on the oocyte retrieval day, regardless of its size. Each follicle was aspirated separately and pooled into a single FF sample from each patient, obtained by combining equal aliquots from two or more FF collection tubes. Each fluid sample was centrifuged at 20000 *g* for 10 min at 4°C to eliminate cells and debris. The supernatant was collected and stored at −80°C until used.

Before oocyte puncture, a peripheral blood sample of each patient was collected by venepuncture of an antebrachial vein into EDTA-containing tubes. The blood was centrifuged at 500 *g* for 5 min at room temperature and the plasma was separated and stored at −80°C.

### 2.4 Hormone analysis of plasma and follicular fluid

Total testosterone, ∆4-androstenedione, progesterone and insulin were measured with an electrochemiluminescence immunoassay (ECLIA, Roche Diagnostics, Mannheim, Germany); sex hormone binding globulin (SHBG) was measured with a chemiluminescent immunometric assay (Immulite XPi instrument, Siemens Healthcare Diagnostics, UK); and Anti-Müllerian hormone (AMH) was measured with an enzyme-linked immunosorbent assay (Beckman Coulter Access AMH, Immunotech, France). All hormone analyses in plasma and FF were performed at the routine core laboratory that serves our centre, based at a public tertiary hospital.

### 2.5 Total antioxidant capacity

The total antioxidant capacity (TAC) was measured by Ferric Reducing Antioxidant Power (FRAP) assay in the FF. FRAP reagent was freshly prepared by mixing 300 mM acetate buffer (pH 3.6), 10 mM 2,4,6-tripyridyl-S-triazine (TPTZ) in 40 mM HCl and 20 mM FeCl3.6H2O (Sigma-Aldrich, St. Louis, Missouri, United States) in the ratio of 10:1:1. Briefly, in a 96-well plate, 6 μL of FF samples were added to 180 µL of FRAP reagent. Triplicates were made for each sample. Distilled water (dH2O) was used as negative control and ascorbic acid (1,000 µM) was used as an antioxidant standard. The absorbance was read on a Synergy™ H1 multi-mode microplate reader (BioTek, Winooski, VT, United States) at OD 593 nm, immediately after the addition of the FRAP reagent (0 min) and after 40 min. Between measurements, the plate was kept in the dark, at room temperature. The antioxidant potential of the samples was determined against the ascorbic acid (1,000 µM) standard and corrected using the absorbance value of dH2O absorbance. FRAP value (µmol/L), was calculated using the formula described in (Benzie and Strain, 1996).

### 2.6 Lipid peroxidation

The lipid peroxidation (LPO) was evaluated by quantifying malondialdehyde (MDA) levels in the FF. LPO was determined using the MDA Assay Kit (ab118970, Abcam Cambridge, MA, United States) according to the manufacturer’s instructions. The MDA that is present in plasma sample reacts with thiobarbituric acid (TBA) to generate an MDA-TBA adduct that is quantified colorimetrically. For the colorimetric assay, a 0.1 mol/L MDA standard was prepared, and serial dilutions were made for the standard curve. Each FF sample and standard was pipetted into a 96-well plate (clear bottom black plate) and colorimetric measurement was taken on a Synergy™ H1 multi-mode microplate reader (BioTek, Winooski, VT, United States) at OD 532 nm. The absorbance value of the blank (water) was used for background subtraction.

### 2.7 Statistical analysis

Results are presented as mean ± standard deviation (SD). The normality of the data was tested according to the Kolmogorov-Smirnov test. Continuous variables were assessed for normal distribution and homogeneity of variance, and the differences between the study groups were evaluated by Student’s t-test for parametric data. In cases where the data did not meet these assumptions (non-normal distribution or heterogeneity of variance), the Mann-Whitney test was employed. The Pearson test or Spearman rank test was used to assess the correlation between two different variables, depending on whether the distribution was normal or non-normal, respectively. The statistical analysis of this work was performed by GraphPad Prism eight software and IBM SPSS Statistics 29.0. Values of *p* < 0.05 were considered as statistically significant.

## 3 Results

### 3.1 Clinical characteristics of study patients

A total of 84 women undergoing IVF were divided into two groups. The anthropometric, clinical and biochemical data of women allocated to the PCOS group (n = 30) and non-PCOS group (n = 54) are shown in Table 1. The study groups did not vary in terms of age, body mass index (BMI), prolactin, thyroid stimulating hormone (TSH), glucose and insulin levels. Circulating levels of follicle-stimulating hormone (FSH) were significantly lower in women with PCOS. Circulating levels of luteinizing hormone (LH) and AMH and LH:FSH ratio were significantly higher in women with PCOS. Hormone levels were also measured in plasma and FF was collected at oocyte retrieval day. Women in the PCOS group had significantly higher plasma levels of ∆4-androstenedione, total testosterone and a higher free androgen index (FAI) than observers in non-PCOS controls. In women with PCOS, FF total testosterone levels were also higher, while there were no significant differences in the other parameters analysed, which were comparable between the study groups.

**TABLE 1:** Demographic and clinical characteristics of women according to study group.

	PCOS (n = 30)	NON-PCOS (n = 54)	*p*-value
Age (years)	32.77 ± 4.04	31.33 ± 5.05	*p* = 0.19
BMI (kg/m^2^)	25.43 ± 5.17	24.11 ± 3.19	*p* = 0.16
Baseline hormone levels			
E_2_ (pg/mL)*	46.52 ± 34.87	46.41 ± 20.96	*p* = 0.24
LH (mIU/mL)*	9.24 ± 3.77	6.25 ± 1.94	*p* < 0.001
FSH (mIU/mL)*	6.54 ± 1.71	7.67 ± 2.04	*p* < 0.05
LH: FSH ratio*	1.49 ± 0.74	0.85 ± 0.28	*p* < 0.001
Prolactin (ng/mL)	19.04 ± 9.80	18.63 ± 11.83	*p* = 0.90
TSH (μIU/mL)	2.02 ± 0.86	1.96 ± 0.79	*p* = 0.58
Glucose (mg/dL)	87.17 ± 11.28	85.18 ± 12.31	*p* = 0.57
Insulin (μIU/mL)	8.66 ± 7.47	5.82 ± 3.85	*p* = 0.19
HOMA-IR	2.00 ± 1.97	1.26 ± 0.87	*p* = 0.26
AMH (pmol/L)	46.93 ± 32.34	22.59 ± 13.95	*p* < 0.0001
Oocyte retrieval day			
p∆4-androstenedione (ng/mL)	4.19 ± 2.26	3.13 ± 1.17	*p* < 0.05
pTestosterone (ng/mL)	1.72 ± 1.23	1.20 ± 0.38	*p* < 0.05
pSHBG (nmol/L)	202.8 ± 111.1	155.5 ± 59.38	*p* = 0.12
pFAI	4.30 ± 3.20	2.63 ± 1.57	*p* < 0.05
ff∆4-androstenedione (ng/mL)	9.39 ± 5.73	8.54 ± 6.54	*p* = 0.27
ffTestosterone (ng/mL)	8.89 ± 6.14	6.34 ± 3.84	*p* < 0.05
ffSHBG (nmol/L)	132.7 ± 47.49	125.8 ± 44.34	*p* = 0.53
ffAMH (pmol/L)	23.04 ± 17.59	20.94 ± 20.26	*p* = 0.19
ffMDA (nmol/L)	23.3 ± 13.51	17.49 ± 7.78	*p* = 0.43
ffFRAP (μmol/L)	170.8 ± 28.20	349.1 ± 77.81	*p* < 0.0001

Values are presented as mean ± standard deviation. *p*-value<0.05 was considered statistically significant. AMH: anti-Müllerian hormone; BMI: body mass index; E_2_: estradiol; FAI: free androgen index; ff: follicular fluid; FSH: follicle-stimulating hormone; FRAP: ferric reducing antioxidant power; HOMA-IR: homeostatic model assessment for insulin resistance; IU: international units; LH: luteinizing hormone; MDA: malondialdehyde; p: plasma; PCOS: polycystic ovary syndrome; SHBG: sex hormone binding globulin; TSH: thyroid-stimulating hormone. *parameters with n = 31 in the non-PCOS group (excluding the oocyte donors).

### 3.2 IVF cycle parameters

The IVF cycle characteristics of PCOS groups (n = 30) and non-PCOS (n = 31, excluding the oocyte donors) are present in Table 2. Among IVF cycle characteristics, the number of cumulus-oocyte complexes (COCs) and the number of two pronucleated oocytes were significantly higher in women with PCOS. There were no significant differences in the other parameters analyzed between groups.

**TABLE 2:** *In vitro* fertilization (IVF) cycle characteristics.

	PCOS (n = 30)	NON-PCOS (n = 54)	*p*-value
Total FSH dose, IU	2,222 ± 508	2,262 ± 662	*p* = 0.78
No. of stimulation days	10.27 ± 1.60	9.78 ± 1.88	*p* = 0.14
No. of COCs	18 ± 9	13 ± 8	*p* < 0.05
No. of two pronucleated oocytes*	7 ± 4	5 ± 4	*p* < 0.05
No. of mature oocytes*	11 ± 6	9 ± 5	*p* = 0.25
Oocyte immaturity rate (%)*	18.34	22.27	*p* = 0.26
Fertilization rate (%)*	65.13	60.00	*p* = 0.17
Cleavage rate (%)*	94.04	97.83	*p* = 0.21
Blastocyst rate (%)*	46.96	54.59	*p* = 0.32

Values are presented as mean ± standard deviation. *p*-value<0.05 was considered statistically significant. COCs: cumulus-oocyte complexes; FSH: follicle-stimulating hormone; IU: international units. *parameters with n = 31 in the non-PCOS group (excluding the oocyte donors). Oocyte immaturity rate: rate of the number of immature oocytes/number of cumulus-oocyte complexes; Fertilization rate: rate of the number of two pronucleated oocytes/number of cumulus-oocyte complexes; Cleavage rate: rate of the number of cleaved embryos/number of two pronucleated oocytes; Blastocyst rate: rate of the number of blastocysts/number of cleaved embryos.

### 3.3 Oxidative stress parameters in the follicular fluid

**FIGURE 1:**
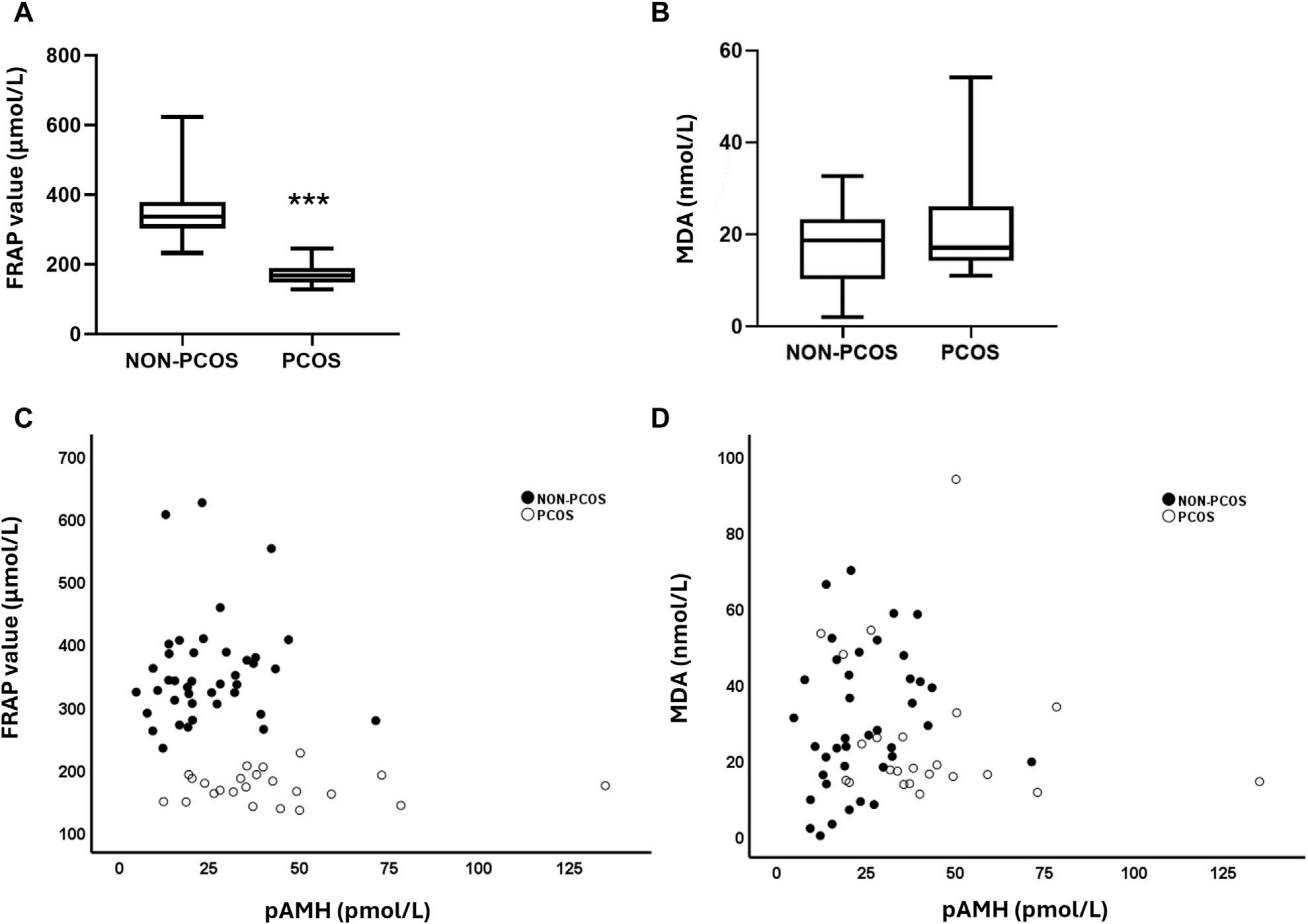
**(A)** Total antioxidant capacity (TAC) levels among non-PCOS vs. PCOS groups. **(B)** Lipid peroxidation (LPO) levels among non-PCOS vs. PCOS groups. **(C)** Correlation between FRAP value and plasma AMH among non-PCOS vs. PCOS groups. **(D)** Correlation between MDA levels and plasma AMH among non-PCOS vs. PCOS groups. FRAP: ferric reducing antioxidant power; MDA: malondialdehyde. Statistical analysis was performed using the Mann-Whitney U test, ***p < 0.0001.

We evaluated the FRAP and the MDA levels in FF of women with PCOS and compared it to those of non-PCOS (Figure 1). We observed significantly lower FRAP levels and a trend for higher MDA levels in the FF of women with PCOS (Table 1).

### 3.4 Relationship between AMH and hormone levels and oxidative stress parameters

To investigate whether there was an association between circulating hormone levels and oxidative stress parameters on the FF, we performed a correlation analysis across our entire population (women with PCOS and without PCOS) (Table 3). A positive correlation was found between plasma AMH and FF AMH, as well as between plasma AMH and plasma ∆4-androstenedione. Additionally, plasma AMH levels were negatively correlated to those of FF progesterone. Moreover, plasma AMH was negatively correlated with FF FRAP levels, while there was no statistically significant correlation between plasma AMH levels and FF LPO (Figures 1C, D; Table 3).

**TABLE 3:** Correlation between serum anti-Müllerian hormone (AMH) and clinical parameters and oxidative stress markers.

	pAMH r (Spearman’s rank correlation coefficient)	*p*-value
p∆4-androstenedione (ng/mL)	0.382	*p* < 0.01
ffProgesterone (ng/mL)	−0.427	*p* < 0.001
ffAMH (pmol/L)	0.403	*p* < 0.001
FRAP (µmol/L)	−0.298	*p* < 0.05
MDA (nmol/mL)	0.027	*p* = 0.84

AMH: anti-Müllerian hormone; ff: follicular fluid; FRAP: ferric reducing antioxidant power; MDA: malondialdehyde; p: plasma.

## 4 Discussion

The finding that AMH in the circulation of women with PCOS is elevated, not only raised the question of whether this could be a biomarker with potential diagnostic applications (Teede et al., 2023), but also prompted significant inquiries into which could be its role in the pathophysiological mechanisms underlying this condition.

PCOS is characterized by gonadotrophic axis dysfunction besides other hormone imbalances. Elevated circulating levels of LH, testosterone and AMH are the hallmarks of this gonadotrophic axis dysregulation. In women with PCOS, the typical gonadotrophin secretion pattern consists of increased LH secretion with normal follicle-stimulating hormone secretion, which results in an abnormal LH/FSH ratio that is considered a valuable marker for evaluating ovarian function while assisting PCOS diagnosis (Dewailly et al., 2020). In this study, as it would have been expected and further supporting the diagnosis, women with PCOS had higher levels of AMH and LH, lower levels of FSH and a higher LH:FSH ratio, when compared to ovulatory controls. Additionally, women with PCOS had significantly higher androgen levels, namely, total testosterone, ∆4-androstenedione, and FAI than women in the control group. Likewise, the total testosterone levels in the FF of women with PCOS were also significantly higher than observed in FF of control women. Remarkably, the presence of elevated androgens is one of the parameters included in the Rotterdam criteria for PCOS diagnosis (Rotterdam ESHRE/ASRM-Sponsored PCOS consensus workshop group, 2004).

Furthermore, we found a positive correlation between circulating levels of AMH and ∆4-androstenedione. While both hormones can be similarly affected in PCOS and the correlation between AMH and ∆4-androstenedione levels has been previously described, the significance of this direct correlation is not well-established (Leonhardt et al., 2014; Piltonen et al., 2023; Barbagallo et al., 2024). Additionally, relationship between AMH and ∆4-androstenedione in different physiological and pathological contexts remains to be elucidated in order to understand whether and how they may influence each other (Piltonen et al., 2019). Notwithstanding, this finding further supports the potential relevance of considering the use of AMH for diagnosing and grading PCOS severity, in line with the growing trend of international groups working towards the refinement of the 2003 Rotterdam criteria (Teede et al., 2023).

In our study, circulating and FF AMH levels were found to be positively correlated, allowing us to reasonably infer that circulating AMH levels reflect the follicular microenvironment in the ovary, lined by the granulosa cell layer responsible for AMH secretion (van Rooij et al., 2002).

The implications of FF AMH levels for follicle physiology are not well-established, nor is the predictive value of FF AMH on reproductive outcomes, particularly in women with PCOS. However, it is recognized that the balance between FSH and AMH within granulosa cells is pivotal in the shift from androgen-to oestrogen-driven follicles (Dewailly et al., 2016) Besides, high levels of AMH in the FF may have harmful consequences for the oocyte quality and final maturation (Desforges-Bullet et al., 2010), as well as fertilization and cleavage rate (Mashiach et al., 2010), although the mechanisms are unknown.

Several studies have explored the relationship between baseline serum AMH levels and pregnancy outcomes following IVF treatment (Liu et al., 2022; Hou et al., 2023; Li et al., 2023; Yuwen et al., 2023). In women with PCOS results have been inconsistent with some authors reporting higher AMH levels to be associated with better reproductive outcomes (Xi et al., 2012; Tal et al., 2015), while others found the opposite (Xi et al., 2012; Tal et al., 2020; Guo et al., 2021; Sun et al., 2021; Vale-Fernandes et al., 2023a). Our results showed that the number of cumulus-oocyte complexes (COCs) and the number of two pronucleated oocytes were significantly higher in women with PCOS. These disparities could be attributed to intrinsic differences between study populations and study population sizes. A previous study conducted by our group uphold the hypothesis that in women with PCOS, high circulating levels of AMH, instead of reflecting the ovarian reserve, act as a biomarker of disease severity and worse reproductive prognosis (Vale-Fernandes et al., 2023a). Overall, the available evidence highlights the unmet need of further understanding the role of AMH in the processes that drive folliculogenesis in the normal and PCOS, which will be key for the treatment of anovulation.

Besides, a negative correlation was found between circulating AMH levels and progesterone levels in the FF. The relationship between AMH and progesterone levels in the FF has not been previously described. While both hormones are involved in reproductive processes, these are secreted by different ovarian cell types and regulated by distinct mechanisms. AMH is primarily involved in ovarian follicle development and is mainly produced by small follicles in the ovaries (van Rooij et al., 2002). Progesterone, on the other hand, is a hormone produced mainly by the corpus luteum after ovulation and plays a crucial role in the menstrual cycle and pregnancy (Chemerinski et al., 2024). In PCOS, AMH levels are often elevated due to an increased number of small ovarian follicles, while progesterone levels may be reduced or irregular due to anovulation or luteal phase defects (Teede et al., 2023). Considering that progesterone is the hormone that highlights ovulation and women with PCOS are predominantly anovulatory, this is an interesting finding not previously described in the literature, that requires greater contextualization and pathophysiological clarification, given that the progesterone measurement was carried out in the context of controlled ovarian hyperstimulation. Additionally, it should be recalled that high progesterone levels at the end of the follicular phase in women undergoing controlled ovarian hyperstimulation for IVF is a reason for suspending fresh embryo transfer and undergoing embryos cryopreservation with subsequent endometrial preparation for transfer of cryopreserved embryos (Bosch et al., 2024).

There is growing evidence suggesting that OS may play a role in the reproductive outcomes of women with PCOS, namely, fertilization and pregnancy rates (Yilmaz et al., 2016; Naigaonkar et al., 2021; Moreira et al., 2023). Studies have indicated that women with PCOS often exhibit higher levels of oxidative stress markers, such as increased ROS production and LPO, and decreased TAC, at the FF compared to women without PCOS (Chattopadhayay et al., 2010; Naigaonkar et al., 2021). In our herein study, we found that the TAC of the FF, as evaluated by FRAP, was significantly lower in women with PCOS as compared to controls. Regarding LPO, although we found a tendency for MDA levels to be higher in the FF of women with PCOS, we did not observe statistically significant differences between women with PCOS and controls. LPO is a consequence of oxidative stress on cell membrane structure and function. This process is characterized by the molecular modification of lipids containing carbon-carbon double bond(s), especially polyunsaturated fatty acids, when exposed to free radicals (Ayala et al., 2014). During LPO different aldehydes can be formed as secondary products, among which MDA and 4-hydroxynonenal (4-HNE) have been widely used as OS markers. However, MDA measures a specific consequence of OS, which could not be particularly affected in this condition and thus explain why no significant differences were observed in our study.

Most importantly, we were able to demonstrate that the higher the circulating AMH and the FF AMH levels, the lower is the TAC. Given that women with PCOS present significantly higher levels of circulating androgens and AMH, in parallel with those observed in the FF, and that these are inversely correlated with the TAC, it seems reasonable enough to raise the hypothesis that AMH could be involved in the processes leading to OS within the FF of women with PCOS, which could then be reflected in poorer reproductive outcomes, as compared to normal ovulatory women particularly during IVF treatments (Vale-Fernandes et al., 2023a).

PCOS is associated with sub-optimal oocyte quality and poor outcomes of ART treatments (Palomba et al., 2017). The evaluation of IVF treatments efficacy still relies mainly on morphological parameters of egg and embryo quality. The redox dynamics in the oocyte microenvironment that encompasses the FF is not assessed nor used in clinical practice in the decision algorithm in ART treatments. However, more and more studies raise the possibility that the study of FF biomarkers could be a fundamental tool to identify the factors that influence the quality of the oocyte/embryo (Naigaonkar et al., 2021; Vale-Fernandes et al., 2023a). By examining the relationship between circulating and FF AMH and TAC, this study provides insights into the potential role of AMH in modulating oxidative stress and its implications for IVF success. Moreover, grounded on AMH levels, it now allows to identify a subset of women with PCOS potentially amenable for intervention to reduce OS aimed at improving the reproductive outcomes.

It is noteworthy to acknowledge that despite the relevant contributions that this study provided, there are several limitations to mention. First of all, this was a single center study and therefore the recruitment capacity was limited by that fact. Second, given the small number of women per group that were enrolled, further stratification of women, with regards to BMI and insulin resistance as assessed by HOMA-IR, were rendered impossible. These would be of particular interest given the well-established association between obesity, insulin resistance and oxidative stress. Notwithstanding the fact that despite the BMI and HOMA-IR tended to be higher in women of the PCOS group compared to the control group these were well balanced between groups and the average did not fall in the obesity nor insulin resistant range. Third, since the circulating levels of AMH in women undergoing IVF treatments are routinely measured between the third to fifth day of the menstrual cycle, up to 6 months before starting the ovarian stimulation, as a surrogate measure of the ovarian reserve, and reassessment of AMH levels at the day of follicular puncture was not conducted, the correlation with FF AMH has to be interpreted taking this limitation into account. And last but not least, since our control group encompassed predominantly healthy women oocyte donors, who did not seek fertility, precluded the possibility of evaluating end stage reproductive parameters, such as pregnancy rates and live births.

## 5 Conclusion

This study results lead us to the reasonable hypothesis of greater susceptibility of the follicle microenvironment to OS being at the end of a roadway that starts with elevated ∆4-androstenedione and AMH within the FF, which in turn are mirrored by circulating AMH and androgen levels that are inversely related to the total anti-oxidant capacity. This end of the spectrum of the hormonal profile is what distinguishes women with and without PCOS, highlighting the central role of androgens and AMH for the phenotype characterization and predicting reproductive outcomes in women affected. The hypothesis that circulating levels of AMH could act as a surrogate biomarker of follicular fluid oxidative stress in PCOS, along with its importance in predicting disease severity and reproductive prognosis in women with PCOS undergoing IVF ttreatments, make the proposal to include AMH in the review of PCOS diagnostic and classification criteria quite pertinent and useful.

## Data Availability

The raw data supporting the conclusions of this article will be made available by the authors, without undue reservation.
